# Evaluation of seroma formation in mesh fixation techniques for inguinal hernia repair

**DOI:** 10.6026/973206300220084

**Published:** 2026-01-31

**Authors:** Kaushlendra Singh Narwariya, Krishna Pratap Singh, Upendra Singh, Pooja Rajput, Vishnu Kumar Gupta, Parul Nema

**Affiliations:** 1 Department of General Surgery, S.R.V.S. Medical College, Shivpuri, Madhya Pradesh, India; 2 Department of Surgery, Motilal Nehru Medical College, Prayagraj, Uttar Pradesh, India; 3 Department of General Surgery, Government Medical College, Satna, Madhya Pradesh, India; 4 Consultant Radiologist, Sai diagnostic Centre and Surgical care, Shivpuri, Madhya Pradesh, India; 5 Department of Community Medicine, SRVS Medical College, Shivpuri, Madhya Pradesh, India; 6 Department of Pathology, SRVS Medical College, Shivpuri, Madhya Pradesh, India

**Keywords:** Inguinal hernia, mesh fixation, seroma, fibrin glue, lichtenstein repair

## Abstract

Seroma formation is a common complication after Lichtenstein tension-free inguinal hernia repair. Therefore, it is of interest to compare the incidence of seroma formation and related postoperative outcomes among non-absorbable sutures, absorbable sutures and fibrin glue fixation techniques. Hence, a total of 180 patients were randomly assigned to three groups for mesh fixation. Fibrin glue fixation resulted in shorter operative times and less postoperative pain but had a higher incidence of seroma formation. Suture-based fixation methods demonstrated fewer seromas and comparable recurrence and complication rates.

## Background:

Mesh-based repair has become the standard approach for inguinal hernia because it provides durable reinforcement of the inguinal canal with low recurrence rates and broad reproducibility. Various mesh fixation strategies are available for both open and laparoscopic approaches and an umbrella review of systematic reviews and meta-analyses has summarized that no single fixation method has proven universally superior with respect to long-term recurrence, so choice is commonly influenced by early postoperative outcomes, cost and surgeon preference [1]. Among early postoperative complications, seroma formation is common and may cause patient discomfort, increased pain scores, delayed recovery and anxiety owing to its resemblance to recurrence; recent investigations have therefore focused on identifying risk factors and the clinical impact of seroma after inguinal hernia repair [2]. Nonpenetrating fixation using biologic adhesives such as fibrin glue has been proposed as an alternative to suture or tack fixation with the dual aims of reducing tissue trauma and lowering postoperative pain. Meta-analyses and randomized studies in the literature indicate that glue fixation is associated with reduced acute and chronic pain and similar short-term morbidity and recurrence rates compared with penetrating fixation, although pooled estimates for seroma and hematoma have been heterogeneous across trials [3].

Several single-centre randomized controlled trials comparing fibrin glue to conventional suture fixation in open Lichtenstein repair have reported shorter operative time and lower pain scores with glue, while differences in seroma incidence have varied between studies, highlighting the need for further controlled comparisons with adequate sample size and standardized outcome assessment [4, 5]. More recent randomized and comparative series continue to report advantages of glue fixation in terms of reduced immediate postoperative pain and shorter hospitalization, but they also emphasize variability in seroma outcomes that may relate to application technique, device characteristics and mesh properties [6,7]. Experimental work has further demonstrated that the method of fibrin application (for example, spray versus sequential layering) significantly affects adhesive strength to the abdominal wall, providing a potential mechanistic explanation for some of the clinical heterogeneity observed between studies [7]. Contemporary prospective clinical studies therefore recommend that evaluation of fixation technique should include standardized definitions for seroma (clinical plus ultrasonographic confirmation), objective measures of resolution time and sufficient follow-up to assess chronic pain and recurrence [8]. Therefore, it is of interest to evaluate seroma formation and related postoperative outcomes following Lichtenstein repair using non-absorbable sutures, absorbable sutures and fibrin glue.

## Materials and Methods:

## Study design and setting: 

This prospective, randomized comparative study was conducted at a tertiary care teaching hospital. Written informed consent was obtained from all participants before inclusion. The objective was to evaluate the incidence of seroma formation following Lichtenstein inguinal hernia repair using different mesh fixation techniques.

## Study population and sample size:

A total of 180 adult male patients diagnosed with primary, unilateral, reducible inguinal hernia were included. The sample size was determined based on the estimated difference in seroma formation among the fixation techniques, assuming an α-error of 0.05 and a study power of 80%. Patients were randomly divided into three groups of 60 each using a computer-generated randomization table. Group A underwent mesh fixation with non-absorbable polypropylene sutures, Group B with absorbable polyglactin sutures and Group C using fibrin glue as the fixation material.

## Eligibility criteria:

Patients aged between 18 and 70 years, who were clinically and ultrasonographically confirmed to have a primary, reducible, unilateral inguinal hernia and were fit for spinal or general anesthesia (ASA grade I or II), were eligible for inclusion. Patients with recurrent or bilateral hernias, irreducible or strangulated hernias, diabetes mellitus, immunosuppressive disorders, or those receiving corticosteroids or anticoagulants were excluded. Individuals with a history of previous lower abdominal surgery were also not considered for the study.

## Preoperative evaluation:

All participants underwent detailed history taking and clinical examination. Routine preoperative investigations included complete blood count, fasting blood sugar, renal function tests and coagulation profile. Ultrasonography of the groin was performed to confirm the diagnosis and exclude any associated pathology. All patients received a standard preoperative antibiotic dose one hour before surgery.

## Surgical technique:

All operations were performed under spinal anesthesia using the standard Lichtenstein tension-free mesh repair technique by experienced surgeons. A 6 x 11 cm polypropylene mesh was used in all cases. The difference among the groups lay in the method of mesh fixation. In Group A, the mesh was secured to the inguinal ligament and conjoint tendon with interrupted polypropylene (2-0) sutures. In Group B, fixation was performed with absorbable polyglactin (2-0) sutures using an identical technique. In Group C, fibrin glue was applied along the mesh margins for adhesion without the use of sutures. The external oblique aponeurosis was closed in layers using absorbable sutures and the skin was approximated with interrupted sutures. A suction drain was not routinely inserted except in cases with significant intraoperative oozing. All patients were encouraged to ambulate on the same day after recovery from anesthesia and were discharged once clinically stable.

## Postoperative follow-up: 

Patients were followed up clinically on postoperative days 3, 7 and 14 and subsequently at 1 and 3 months after surgery. Each visit included assessment for seroma formation, wound infection, hematoma, pain and recurrence. Seroma was defined as a clinically detectable, fluctuant, non-tender swelling at the operative site, which was confirmed by ultrasonography when necessary. The volume of seroma, when present, was graded as mild (<10 mL), moderate (10-50 mL), or severe (>50 mL).

## Statistical analysis:

All data were compiled in Microsoft Excel and analyzed using Statistical Package for the Social Sciences (SPSS) version 25. Quantitative variables were expressed as mean ± standard deviation and compared using one-way analysis of variance (ANOVA). Categorical variables were presented as frequencies and percentages and intergroup comparisons were made using the Chi-square test or Fisher's exact test, as applicable. A p-value of less than 0.05 was considered statistically significant.

## Results:

A total of 180 patients undergoing Lichtenstein tension-free hernioplasty were included in the final analysis, with 60 participants in each of the three groups. All groups were comparable in terms of baseline demographic and clinical characteristics such as age, body mass index and hernia type, indicating that randomization was effective Table 1. Operative time, however, showed a significant difference, being shortest in the fibrin glue fixation group, reflecting the technical ease and reduced handling time associated with the sutureless approach. Seroma formation was the most frequent postoperative complication observed across the groups Table 2. The overall incidence differed significantly, being highest in the fibrin glue group, followed by the non-absorbable and absorbable suture groups. Although most seromas were mild and self-limiting, their mean duration of resolution was notably longer in patients who received fibrin glue fixation. This suggests that the absence of tissue penetration with sutures might influence local inflammatory response and lymphatic sealing, contributing to transient fluid collection. Postoperative pain and wound outcomes are summarized in Table 3. Patients in the fibrin glue group experienced substantially lower pain scores during the immediate postoperative period, consistent with reduced tissue trauma from the absence of suturing. The duration of hospital stay also correlated with pain intensity, being shortest in the glue fixation group. The rates of wound infection and hematoma were low and comparable among all groups, indicating that the type of fixation material did not affect wound healing. Follow-up evaluation at three months revealed no statistically significant differences in long-term outcomes, as shown in Table 4. Seroma recurrence and chronic groin pain were infrequent and evenly distributed across the groups. Only one case of hernia recurrence was recorded in the fibrin glue group, which was not statistically significant. These findings suggest that while the fixation technique may influence early postoperative parameters such as operative duration, pain and seroma formation, the long-term success and safety of the procedure remain equivalent across methods.

## Discussion:

This randomized comparative study evaluated seroma formation and other early postoperative outcomes following Lichtenstein inguinal hernia repair using three different mesh fixation techniques. Our results demonstrate a higher incidence and longer resolution time for seroma in the fibrin glue group, accompanied by shorter operative time and lower immediate postoperative pain compared with suture-based fixation. These findings align with the broader literature indicating a trade-off between reduced tissue trauma and an increased tendency for transient fluid collections when non penetrating fixation methods are used [9, 10-11]. Multiple randomized trials and meta-analyses have consistently shown that glue or sutureless fixation reduces operative time and early postoperative pain when compared with suture or tack fixation in open and laparoscopic hernia repairs, which likely reflects decreased tissue handling and fewer penetrative fixation points. The observed reduction in mean operative time and lower early pain scores in the fibrin glue group of our series are concordant with pooled analyses and prospectively collected trial data [9,10, -11]. These advantages can be clinically meaningful in high-volume settings and for ambulatory care pathways where shorter procedures and faster recovery facilitate early discharge. However, the relationship between fixation method and seroma formation is less uniform across studies. While some meta-analyses and RCTs report no significant difference in seroma rates between glue and suture fixation, other trials have documented higher seroma incidence following glue application or variable results depending on glue type and technique of application. In our cohort, the fibrin glue group had a statistically higher seroma rate and longer mean time to resolution, a pattern that may reflect altered local tissue apposition and lymphatic sealing when sutures are not used to compress dead space. Recent systematic reviews emphasize heterogeneity in seroma outcomes and highlight that device characteristics, application technique (spray versus layered) and mesh porosity materially influence fluid accumulation [12, 13-14]. Mechanistic and experimental works support this clinical heterogeneity. *Ex vivo* and *in vivo* experiments show that adhesive strength and the capacity to obliterate dead space depend on how fibrin sealants are delivered and the interaction between sealant and mesh type; inadequate adhesive coverage or differing mesh-glue interfaces can leave potential spaces for seroma formation despite secure overall fixation. Such laboratory observations offer plausible explanations for why some clinical series report low seroma rates with glue while others-like ours-find a higher incidence. These technical nuances underscore the need for standardization of glue application in clinical trials and routine practice [15, 16]. Beyond early seroma, our data show that wound infection, hematoma and three-month recurrence rates were similar across fixation modalities, supporting the view that suture, absorbable suture and glue fixation are broadly comparable in terms of safety and short-term durability. These results are compatible with larger comparative studies and systematic reviews that find no clinically meaningful difference in recurrence between fixation techniques when follow-up is adequate [17, 18]. The near-equivalence of long-term outcomes reinforces that the primary differences among fixation methods concern early postoperative morbidity and patient comfort rather than structural failure of the repair.

The clinical implications of these findings are pragmatic. Surgeons who prioritize shorter operative time and lower immediate postoperative pain-for example in ambulatory settings or in patients at higher risk from prolonged anesthesia-may reasonably consider fibrin glue fixation, recognizing the trade-off of a higher, usually self-limiting, seroma risk. Conversely, in patients where avoidance of any early fluid collection is especially desirable (for instance, those with prior wound healing concerns or where postoperative access to ultrasonography is limited), suture fixation may remain preferable. In both scenarios, surgeon experience with the chosen fixation technique and careful intraoperative attention to obliterating dead space and haemostasis are likely to attenuate seroma risk. This study has several limitations that merit acknowledgement. Although the sample size was adequate to detect the observed differences in seroma incidence, the follow-up period was limited to three months for clinical endpoints; longer surveillance would better characterize late recurrences and persistent chronic pain. Additionally, while fixation techniques were standardized, subtle variations in glue application and mesh handling by different surgeons may have contributed to outcome variability-an inherent challenge in pragmatic surgical trials. Finally, our study was performed in a single tertiary centre and results may not generalize to different healthcare settings or to laparoscopic approaches where fixation dynamics differ.

## Conclusion:

All three mesh fixation techniques such as non-absorbable suture, absorbable suture and fibrin glue are safe and effective for Lichtenstein inguinal hernia repair. Fibrin glue reduced operative time and postoperative pain but was associated with a higher incidence and longer duration of seroma formation. No significant differences were observed in chronic pain or hernia recurrence across the groups during follow-up.

## Figures and Tables

**Table 1:** Baseline demographic and clinical characteristics

**Parameter**	**Group A (Polypropylene) n=60**	**Group B (Polyglactin) n=60**	**Group C (Fibrin Glue) n=60**	**p-value**
Mean age (years) ± SD	46.8 ± 10.2	47.6 ± 9.8	45.9 ± 10.6	0.62
BMI (kg/m^2^) ± SD	24.7 ± 2.3	24.3 ± 2.1	24.6 ± 2.5	0.73
Right-sided hernia (%)	65	68.3	63.3	0.81
Indirect hernia (%)	73.3	76.7	71.7	0.79
Mean operative time (minutes) ± SD	59.4 ± 8.2	57.8 ± 7.9	49.6 ± 6.8	<0.001*

**Table 2:** Incidence and severity of seroma formation

**Seroma Parameters**	**Group A (Polypropylene) n=60**	**Group B (Polyglactin) n=60**	**Group C (Fibrin Glue) n=60**	**p-value**
Seroma formation (%)	10 (16.7%)	8 (13.3%)	18 (30.0%)	0.03*
Mild (<10 mL)	6 (10.0%)	5 (8.3%)	8 (13.3%)	0.41
Moderate (10-50 mL)	3 (5.0%)	2 (3.3%)	7 (11.7%)	0.09
Severe (>50 mL)	1 (1.7%)	1 (1.7%)	3 (5.0%)	0.37
Mean duration of seroma resolution (days) ± SD	10.4 ± 2.8	9.8 ± 2.5	12.1 ± 3.4	0.02*

**Table 3:** Postoperative pain and wound complications

**Parameter**	**Group A (Polypropylene) n=60**	**Group B (Polyglactin) n=60**	**Group C (Fibrin Glue) n=60**	**p-value**
Mean pain score (VAS Day 1) ± SD	5.6 ± 1.1	5.2 ± 1.0	3.8 ± 0.9	<0.001*
Wound infection (%)	3 (5.0%)	2 (3.3%)	2 (3.3%)	0.84
Hematoma (%)	2 (3.3%)	1 (1.7%)	2 (3.3%)	0.79
Mean hospital stay (days) ± SD	3.6 ± 0.8	3.5 ± 0.7	2.8 ± 0.6	<0.001*

**Table 4:** Follow-up outcomes

**Parameter**	**Group A (Polypropylene) n=60**	**Group B (Polyglactin) n=60**	**Group C (Fibrin Glue) n=60**	**p-value**
Seroma recurrence at 3 months (%)	1 (1.7%)	1 (1.7%)	2 (3.3%)	0.79
Chronic groin pain at 3 months (%)	5 (8.3%)	3 (5.0%)	2 (3.3%)	0.37
Recurrence of hernia (%)	0 (0%)	0 (0%)	1 (1.7%)	0.36
